# Supplementary material for: Ultrasound Lung Aeration Map via Physics-Aware Neural Operators
Source: ArXiv. 2025 Jan 2:arXiv:2501.01157v1. Preprint. [Version 1] (PMC11722513)
Supplement: Supplement 1 [file NIHPP2501.01157v1-supplement-1.pdf]

Supplementary Information

Appendix A Data Generation and Acquisition Details

This section describes the creation of lung histology maps for simulating lung ultrasound data. LUNA is trained on abundant simulation data.

Acoustical property maps were generated by combining human chest wall anatomical images (Visible Human Project, 330 μm resolution) with high-resolution histological images of healthy swine lung tissue (5 μm thick, 0.55 μm resolution), following [31]. Binary-segmented histological images quantified lung aeration (air vs. non-air), producing maps where 1 represents air and 0 represents non-air ($\rho^A : \mathbb{Z}^2 \to 0, 1)$. Details of this process are shown in fig. A1 along with the simulation data generation and real data acquisition details.

In fig. A2, we provide the distribution and visualizations of the synthetic and real data used to train and fine-tune LUNA. The small gap between the two data set allows the robustness of LUNA's performance on both synthetic and real data. In fig. A3, we demonstrate that beamforming parameters increase the variation and reduce the reliability of interpreting B-mode images. Thus, machine learning models that take B-mode images as input [20–22] may not generalize well to different ultrasound devices as each device has a setting with different imaging parameters (dynamic range, time gain compensation).

Appendix B Chest Wall Segmentation Results

This section presents the results of chest wall segmentation, highlighting the separation line (pleural line) between the chest wall and the lung, which is consistently located below the chest wall in the input B-mode ultrasound images (fig. B4). For visualization, the separation line is overlaid onto the B-mode images to demonstrate the segmentation performance. On the in silico test set, our model achieves an impressive Dice similarity coefficient [66] of 95.8%, indicating near-perfect alignment with the ground truth segmentation obtained from the simulation. In the ex vivo test set, the model performs reliably despite the inherent domain differences between simulation and real-world data. The results suggest a minimal performance gap between the in silico and ex vivo conditions, demonstrating the robustness of our method.

Appendix C Ablation Study on Network Design

In this ablation study, we investigate the impact of different components of our proposed model on the ex vivo percent aeration prediction performance.

1. **Temporal augmentation**. As discussed in Section 4.3 of the main paper, temporal masking strengthens the invariance of LUNA to temporal delay. Empirically, removing temporal augmentation leads to 6.2% performance loss.
2. **Spatial augmentation**. As discussed in Section 4.3 of the main paper, spatial masking improves LUNA's ability to learn lateral correspondence. Empirically, removing spatial augmentation leads to 0.9% performance loss.
3. **FNO module**. The inclusion of the temporal Fourier Neural Operator (FNO) significantly reduces prediction error compared to replacing it with a ResNet module with equivalent parameters, highlighting the effectiveness of FNO in capturing temporal dynamics. Empirically, replacing FNO with ResNet leads to 1.2% performance loss.
4. **The percent aeration loss**. We set the aeration loss's weight $\eta = 0$ in Eqn. 19. We show that $\mathcal{L}_\gamma$ further refines the model's predictions by directly optimizing the percent aeration accuracy. Empirically, removing percent aeration loss leads to 2.1% performance loss.

These components collectively contribute to the performance improvement as shown in fig. C5.

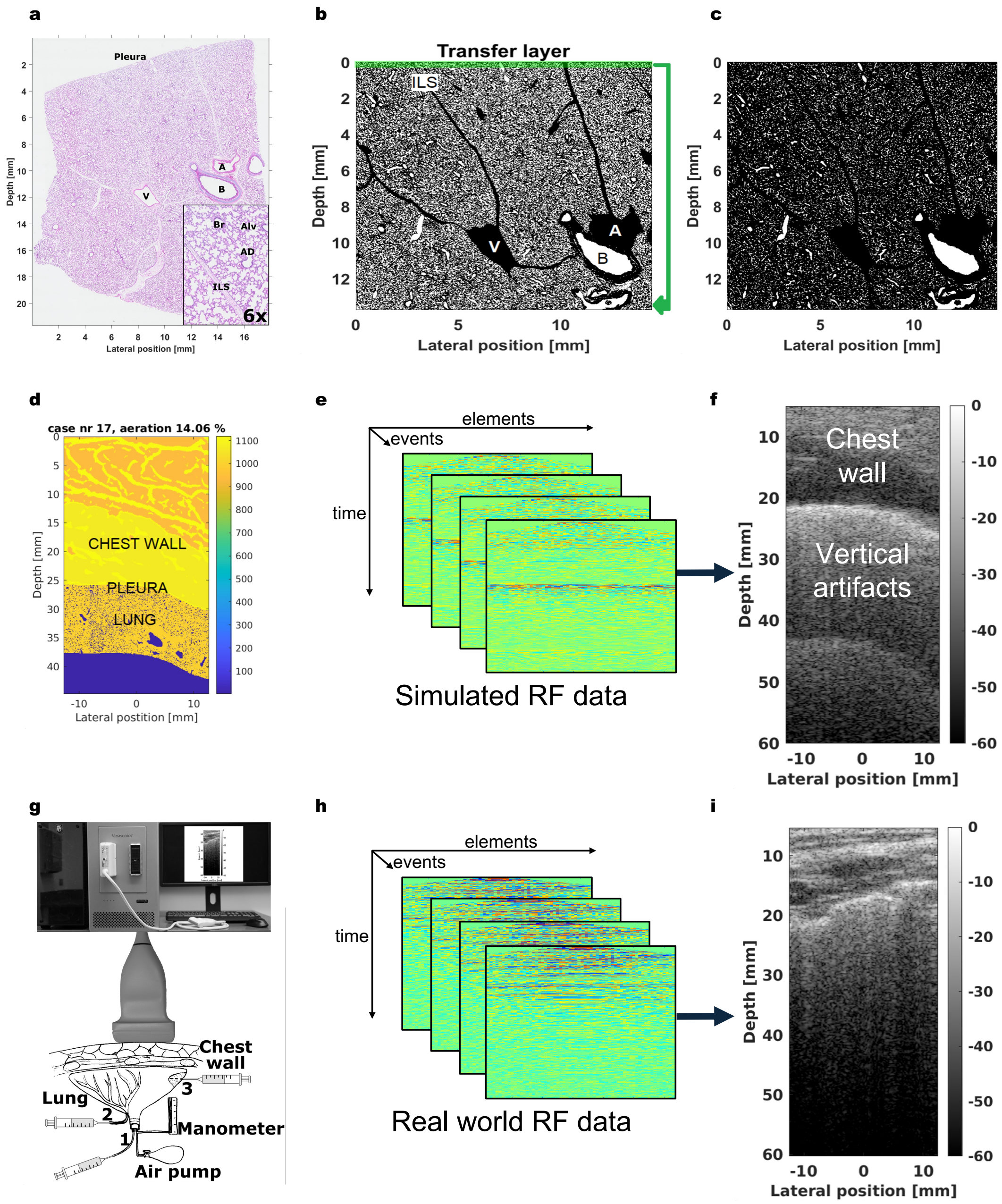

Fig. A1: **The process of ultrasound imaging *in silico* (a-f) and *ex vivo* (g-i).** LUNA is trained on 10k in silico (simulated) data and fine-tuned on 18 ex vivo (real) data. **a,** Formation of the acoustical maps as a stationary input for Fullwave-2 simulation tool: histology (H&E) of healthy swine lung. Insertion shows a magnified part of it and the ability to identify lung tissue architecture. B - bronchus, Br - bronchiole, A - artery, V - vein, AD - alveolar duct, Alv - alveolus, ILS - interlobular septum. **b,** Binary-segmented and leveled histology image deformed to conform to linear pleural line and cropped to a rectangular shape. Its superficial 0.1 mm thick layer (green) is used for repetitive transfer procedures to increase the spatial variability of lung structures. **c,** Segmented histology after applied algorithmic modification modeling ARDS (added fluid/non-air pixels are evenly distributed among tissue-air interfaces) with target aeration of 14%. **d,** Combined aeration map comprised of the tissue-specifically segmented body wall (top) and underlying lung deformed to conform its internal surface which models realistic pleural interface. **e,** Stack of numerically simulated raw RF data of 128 transmit-receive events visualized as the amplitude of received backscattered signal in form receiver-time. **f,** Corresponding B-mode image formed using simulated RF data demonstrates its proper anatomical part (chest wall thickness and composition) along with multiple coalescent vertical artifacts below the pleural line which are consistent with modeled uniformly distributed fluid retention in lung parenchyma. **g,** Scanning of fresh porcine lungs of known aeration (displacement method) through chest wall fragment in the water tank (*ex vivo*) using a programmable ultrasound machine and linear transducer. **h,** Stack of real-world raw RF data of 128 transmit-receive events. **i,** B-mode image formed using real-world RF data demonstrates both anatomical (chest wall tissue, pleural line) and artifactual (multiple coalescent vertical artifacts below the pleural line) parts of the image consistent with modeled significant uniform fluid retention in the lung.

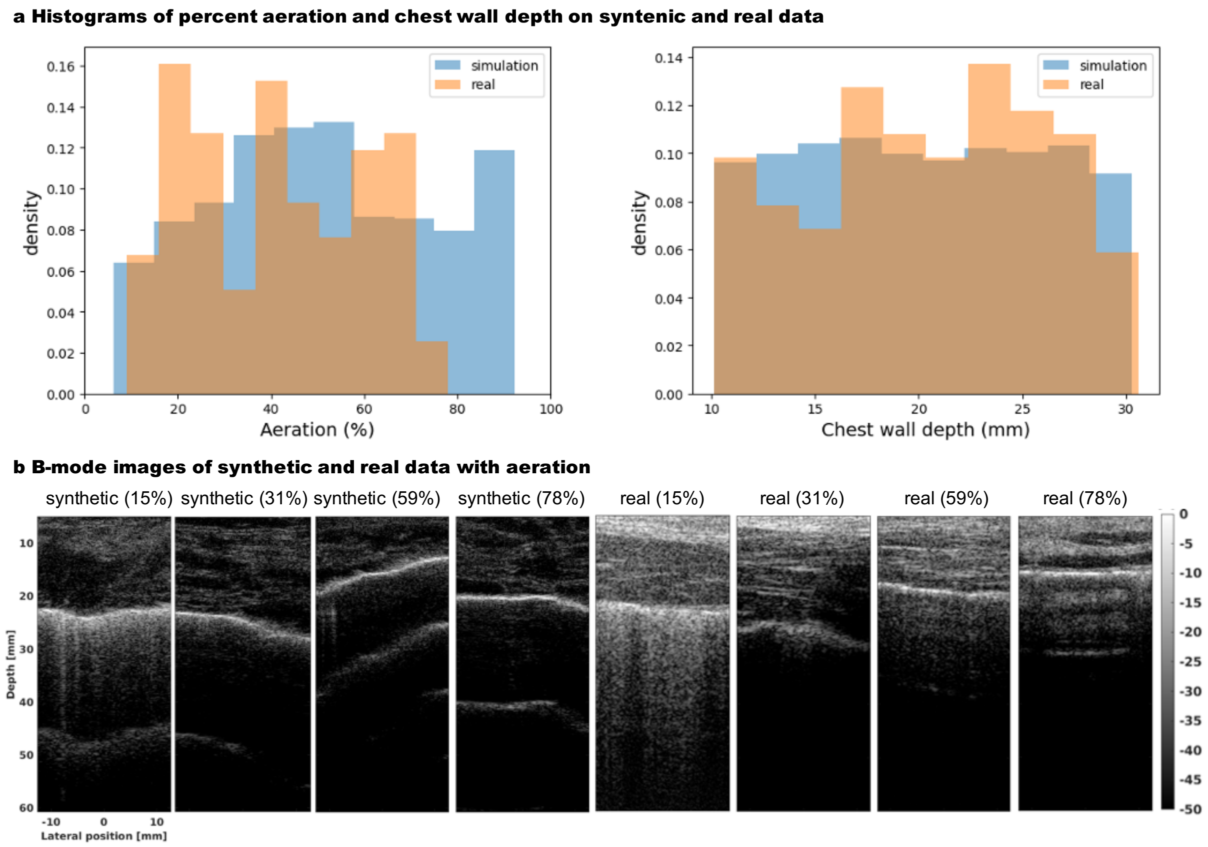

Fig. A2: **The distribution and visualizations of the synthetic and real data used to train and fine-tune LUNA. a,** The histogram of percent aeration and chest wall depth, two important lung properties, of *in silico* and *ex vivo* data. *In silico* data covers all possible lung properties of *ex vivo* data. **b,** B-mode images corresponding to varying levels of aeration used in *in silico* and *ex vivo* experiments, demonstrating a small domain discrepancy between the two datasets.

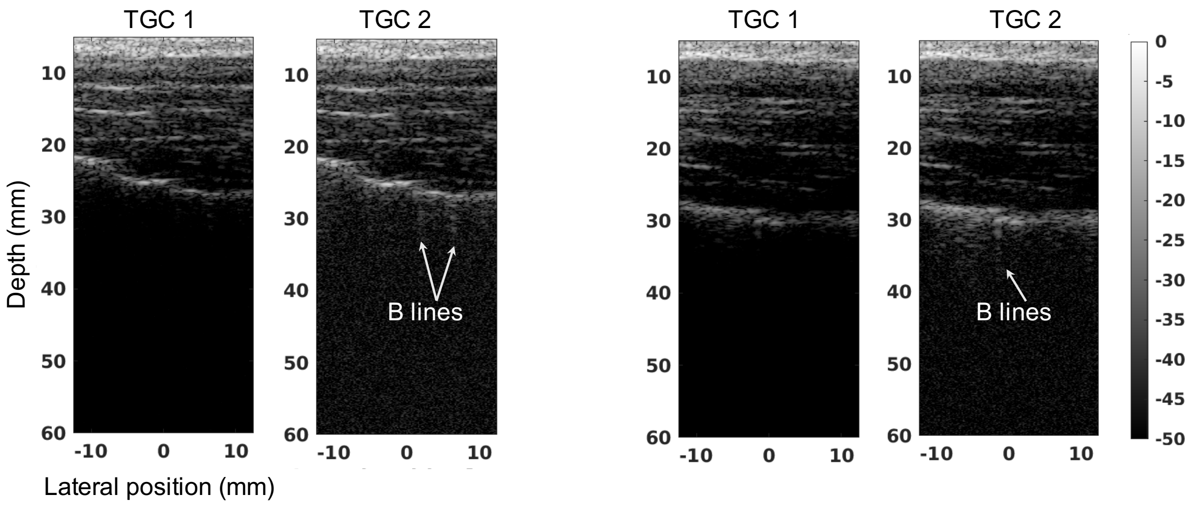

Fig. A3: **Different beamforming parameters increase the variation and reduce the reliability of interpreting B-mode images.** Time gain compensation (TGC), a beamforming parameter, changes the B-mode image artifacts and increases the variability for interpreting the lung status. We show the same sample with two different TGC settings, where under the latter settings artifacts like B-lines are visible. The appearance of B-lines confuses human radiologists and machine learning models that take B-mode images as input, as such artifacts are indicators for low aeration. The left sample has 39% aeration and the right sample has 29% aeration.

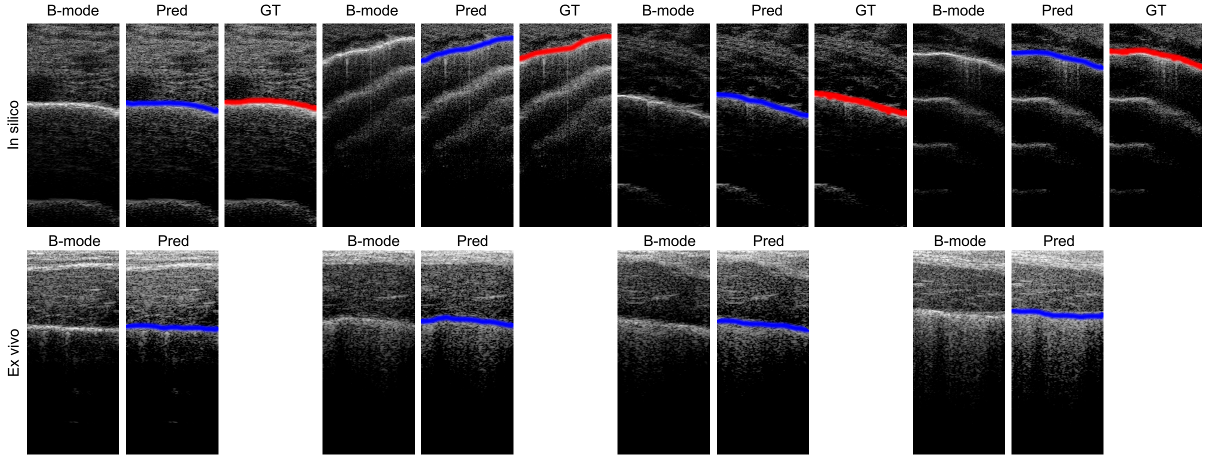

Fig. B4: **Chest wall segmentation results.** The separation line (pleural line) between the chest wall and the lung is overlaid onto B-mode ultrasound images for visualization. Upper: For in silico evaluation samples, the predicted segmentation (Pred) aligns closely with the ground truth (GT) obtained from the simulation. Lower: For ex vivo evaluation samples, the segmentation performance remains robust, with a small performance gap observed between the simulated and real-world datasets.

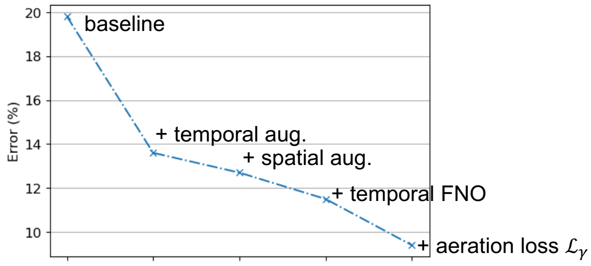

Fig. C5: **Ablation study results of LUNA.** The ex vivo percent aeration prediction performance is shown as different components of the proposed model are progressively added. Temporal augmentation and spatial augmentation improve generalization, while the temporal Fourier Neural Operator (FNO) outperforms a ResNet module with equivalent parameters. The addition of aeration loss $\mathcal{L}_\gamma$ further refines predictions, achieving the lowest error.
